# Transcriptome Analysis Reveals Dynamic Cultivar-Dependent Patterns of Gene Expression in Potato Spindle Tuber Viroid-Infected Pepper

**DOI:** 10.3390/plants10122687

**Published:** 2021-12-07

**Authors:** Nikol Hadjieva, Elena Apostolova, Vesselin Baev, Galina Yahubyan, Mariyana Gozmanova

**Affiliations:** Department of Plant Physiology and Molecular Biology, University of Plovdiv, 24 Tzar Asen Str., 4000 Plovdiv, Bulgaria; nik.hadjieva@abv.bg (N.H.); eapostolova@uni-plovdiv.bg (E.A.); baev@uni-plovdiv.bg (V.B.); gyahubyan@uni-plovdiv.bg (G.Y.)

**Keywords:** PSTVd pathogenicity, RNA-seq, expression analysis, pepper cultivars

## Abstract

Potato spindle tuber viroid (PSTVd) infects various plants. PSTVd pathogenesis is associated with interference with the cellular metabolism and defense signaling pathways via direct interaction with host factors or via the transcriptional or post-transcriptional modulation of gene expression. To better understand host defense mechanisms to PSTVd infection, we analyzed the gene expression in two pepper cultivars, Capsicum annuum Kurtovska kapia (KK) and Djulunska shipka (DS), which exhibit mild symptoms of PSTVd infection. Deep sequencing-based transcriptome analysis revealed differential gene expression upon infection, with some genes displaying contrasting expression patterns in KK and DS plants. More genes were downregulated in DS plants upon infection than in KK plants, which could underlie the more severe symptoms seen in DS plants. Gene ontology enrichment analysis revealed that most of the downregulated differentially expressed genes in both cultivars were enriched in the gene ontology term photosynthesis. The genes upregulated in DS plants fell in the biological process of gene ontology term defense response. We validated the expression of six overlapping differentially expressed genes that are involved in photosynthesis, plant hormone signaling, and defense pathways by quantitative polymerase chain reaction. The observed differences in the responses of the two cultivars to PSTVd infection expand the understanding of the fine-tuning of plant gene expression that is needed to overcome the infection.

## 1. Introduction

Viroids are pathogenic non-coding RNAs, 250–400 nt in length, which infect higher plants [1,2,3]. The sequence and structure of viroids determine their interactions with host factors, resulting in successful replication, spread, and disease induction [4,5,6].

Two viroid families are recognized, *Pospiviroidae* and *Avsunviroidae*, and are distinguished by their structural conformation, cell localization, replication mode, and host range [7]. Members of the genus *Pospiviroid* within the family *Pospiviroidae* mainly infect *Solanaceae* species [8]. Various viroid species/strain and host species/cultivar combinations lead to asymptomatic or symptomatic infections [9,10]. The most recognizable symptoms of viroid infection in plants are stunting, epinasty, chlorosis, necrosis, and the malformation of the reproductive organs [11]. At the cellular level, distortions in the cell membrane and cell wall organization as well as impairment in chloroplast biogenesis and development are the most common symptoms of viroid infection [12].

Potato spindle tuber viroid (PSTVd) is the type member species of the family *Pospiviroidae*. The genome has a characteristic rod-like structure and is 359–361 nt long [2]. It is divided into internal domains—central (C), variable (V), pathogenic (P), terminal left (TL), and terminal right (TR)—which carry out more than one function [4]. PSTVd replicates in the host nucleus by the asymmetric rolling-circle mechanism using cellular DNA-dependent RNA polymerase complexed with a TFIIIA 7ZF transcription factor to adopt PSTVd RNA as a template [13]. PSTVd infection is associated with direct viroid interactions with host factors and the modulation of host gene expression, leading to the dysregulation of host functions [14,15,16]. The activation of plant innate immune responses, alteration of alternative splicing, and interference with the host translational machinery may affect host gene expression [17,18,19,20,21,22]. Symptoms developed in some *Solanaceae* plants during PSTVd infection were accompanied by specific patterns of PSTVd-derived sRNAs and miRNA expression, which collectively affect host gene silencing [23,24,25,26]. PSTVd infection was found to impact the expression of long non-coding RNAs, which act as potent regulatory molecules that modulate the expression of adjacent genes [17].

Transcriptional profiling by high-throughput RNA sequencing (RNA-seq) or microarray analysis in *Solanaceae* species revealed that PSTVd infection has a global effect on host gene expression [27,28,29]. Plant genes altered during PSTVd infection are related to the cell wall structure, chloroplast function, and hormone signaling, which collectively mediate the defense response. Unlike tomato, the symptoms of PSTVd infection in pepper are mild and develop during late stages of infection [30,31]. Recently, the PSTVd infection of two Bulgarian pepper cultivars was reported to be linked to cultivar-specific microRNA (miRNA) dynamics [31]. We analyzed the whole-genome transcriptional responses of two pepper cultivars, Capsicum annuun Djulunska shipka and Kurtovska kapia, to PSTVd infection. In our previous study, the two cultivars showed PSTVd-specific phenotypes, which was more pronounced in DS [31]. The analysis revealed cultivar-specific gene expression patterns in response to viroid infection, with some genes displaying contrasting expressions in the two cultivars. We used gene ontology (GO) enrichment analysis to evaluate the function of the identified differentially expressed genes (DEGs) and their correspondence to specific biological pathways in the PSTVd response in pepper.

## 2. Results

In the current study, the two studied cultivars developed PSTVd-induced phenotypes at 43 days post-inoculation (dpi), with disease symptoms (leaf curling and malformation) being more pronounced in DSI than in KKI [31]. The PSTVd infection was confirmed using strand-specific reverse-transcription polymerase chain reaction (RT-PCR) (Figure S1). The relative accumulation of the PSTVd (+) strand in the two cultivars, as determined by semi-quantitative RT-PCR, showed a slight increase in the relative levels of PSTVd in DSI compared to KKI (Figure S2).

### 2.1. Analysis of RNA-Seq Data

The RNA-seq analysis generated over 173 M reads. The statistics for the raw and clean reads obtained from each sample are presented in Table 1.

**Figure 1:**
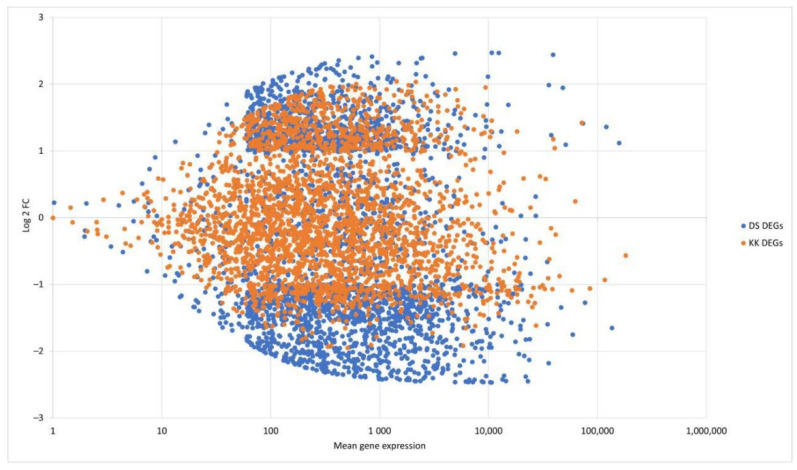
Up- and downregulated genes in KK and DS pepper cultivars in response to PSTVd infection at 43 dpi.

Using DESeq2, we performed pairwise comparisons of the mock and PSTVd-infected plants [32]. The analysis revealed 998 DEGs (43.5% downregulated and 56.5% upregulated) in KK plants and 2161 DEGs (62.3% downregulated and 37.7% upregulated) in DS plants (Figure 1, Table S1).

**Figure 2:**
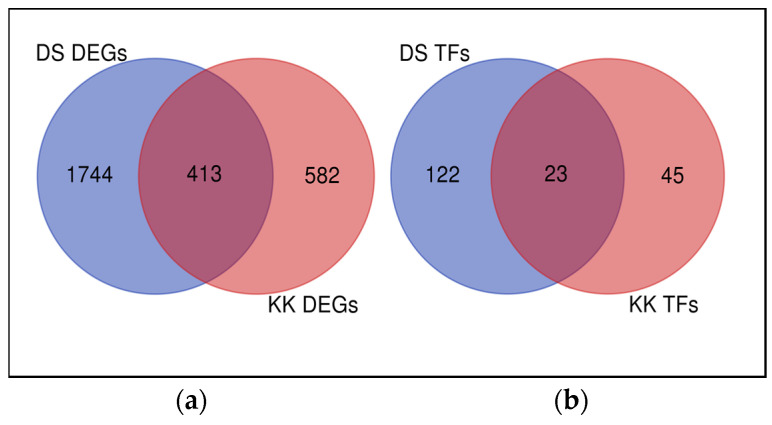
Venn diagrams showing DEGs common or specific to the two pepper cultivars. (**a**) Number of total DEGs, (**b**) number of DE TFs (PlantRegMap/PlantTFDB v5.0). The DEGs were identified in response to PSTVd infection at 43 dpi.

**Figure 3:**
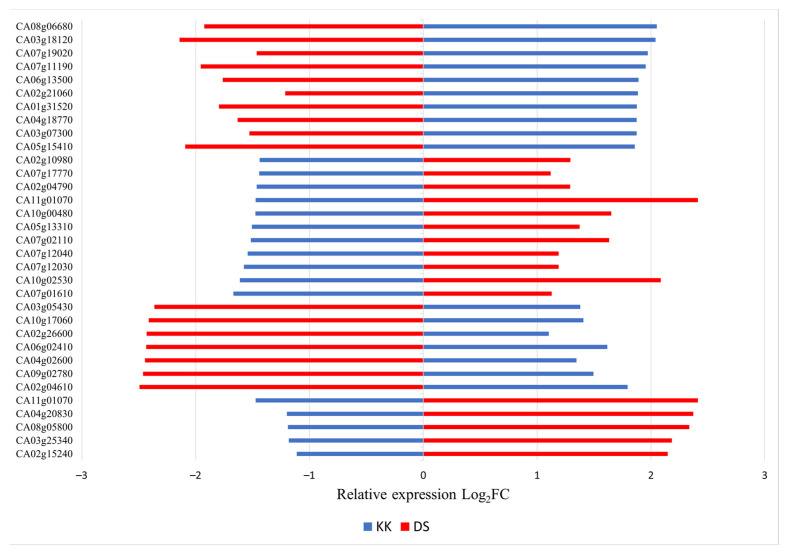
Genes with inverse expression patterns in two pepper cultivars in response to PSTVd at 43 dpi, identified by RNA seq. The comparison of the expression levels of mock (KKH, DSH) and PSTVd infected (KKI, DSI) was performed using the DESeq2 package.

The expression of twice as many genes was affected by viroid infection in DS than in KK (Figure 2a). A total of 1346 genes were downregulated in DS; by contrast, 434 genes in KK exhibited a decline in expression. In total, 815 genes and 564 genes were upregulated in DS and KK, respectively. A total of 413 overlapping DEGs were identified in the two cultivars, and the number of DS-specific DEGs was threefold greater than that of KK-specific DEGs (Figure 2a). Of the 413 common DEGs, 224 showed opposite expression patterns in the two cultivars (Figure 3).

Among the overlapping DEGs, we identified 23 transcription factors (DE TFs), most of which showed an expression change in the opposite direction (Figure 2b, Table S2). Most of the DS-specific DE TFs were downregulated, whereas most of the KK-specific DE TFs were upregulated.

### 2.2. Functional Annotation of DEGs

**Figure 4:**
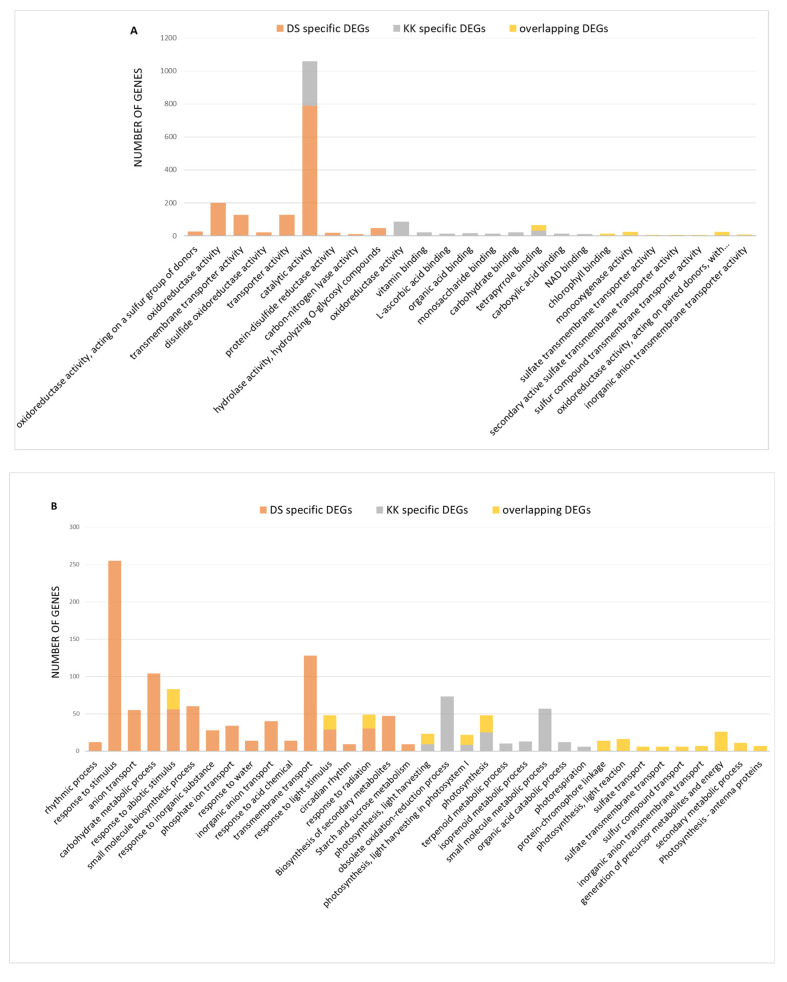

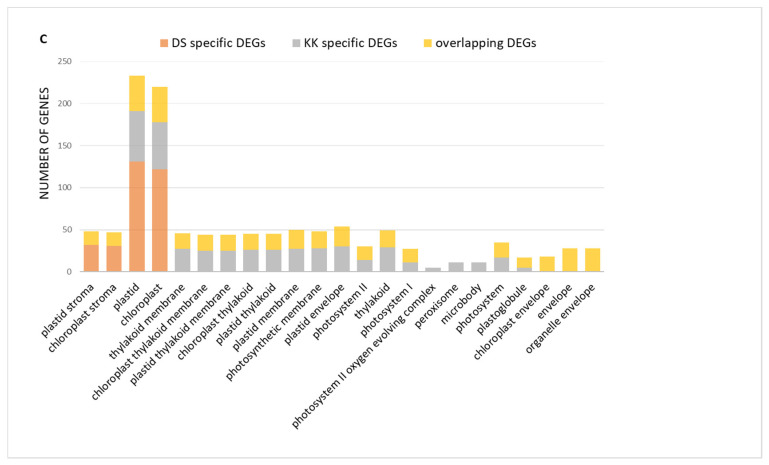
Gene ontology enrichment analysis of DEGs in the two pepper cultivars. GO terms enriched in: molecular function (**A**), biological process (**B**), and cellular component (**C**) across DS-specific, KK-specific, and overlapping DEGs using g: Profiler (adj *p* < 0.05).

The GO enrichment analysis of the overlapping and cultivar-specific DEGs using g: Profiler was performed to link DEGs with specific biological processes (BP), molecular function (MF), and cellular components (CC) (Table S3). The overlapping DEGs included: 8 MF GO terms, the most enriched of which were chlorophyll binding, tetrapyrrole binding, oxidoreductase, and monooxygenase activity; 20 CC GO terms, including plastid, chloroplast, envelop, organelle envelop, plastoglobule, photosystem I and II; and 14 BP GO terms, including photosynthesis, light harvesting in photosystem I, the generation of precursor metabolites and energy, response to stimulus, and transport (Figure 4, Table S3). DS-specific DEGs included: 9 MF GO terms, such as catalytic, oxidoreductase, and transmembrane transporter activity; 4 CC GO terms, among which plastid and chloroplast were predominant; and 17 BP GO terms, the most enriched of which were response to stimulus, transmembrane transport, carbohydrate metabolic process, small molecule biosynthesis process, the biosynthesis of secondary metabolites, and starch and sucrose metabolites (Figure 4, Table S3). KK-specific DEGs included 10 MF GO terms, such as catalytic, oxidoreductase activity, vitamin binding, tetrapyrrole binding; 18 CC GO terms, the most enriched of which were plastid, chloroplast, plastid envelope, and thylakoid membrane; and 9 BP GO terms, including obsolete oxidation–reduction processes, small molecule metabolic process, photosynthesis, and photorespiration (Figure 4, Table S3). In both cultivars, the downregulated genes were enriched in the BP GO terms photosynthesis and response to stimulus. On the other hand, the genes upregulated in response to PSTVd infection in DS plants were enriched in the BP GO term defense response, while those upregulated in KK plants were enriched in the BP GO terms obsolete oxidoreductase processes and response to stimulus (Table S4). Furthermore, the enriched GO terms were summarized with REVIGO (Figure S3).

### 2.3. Comparative Expression Analysis of Overlapping DEGs in Two Pepper Cultivars Infected with PSTVd

**Figure 5:**
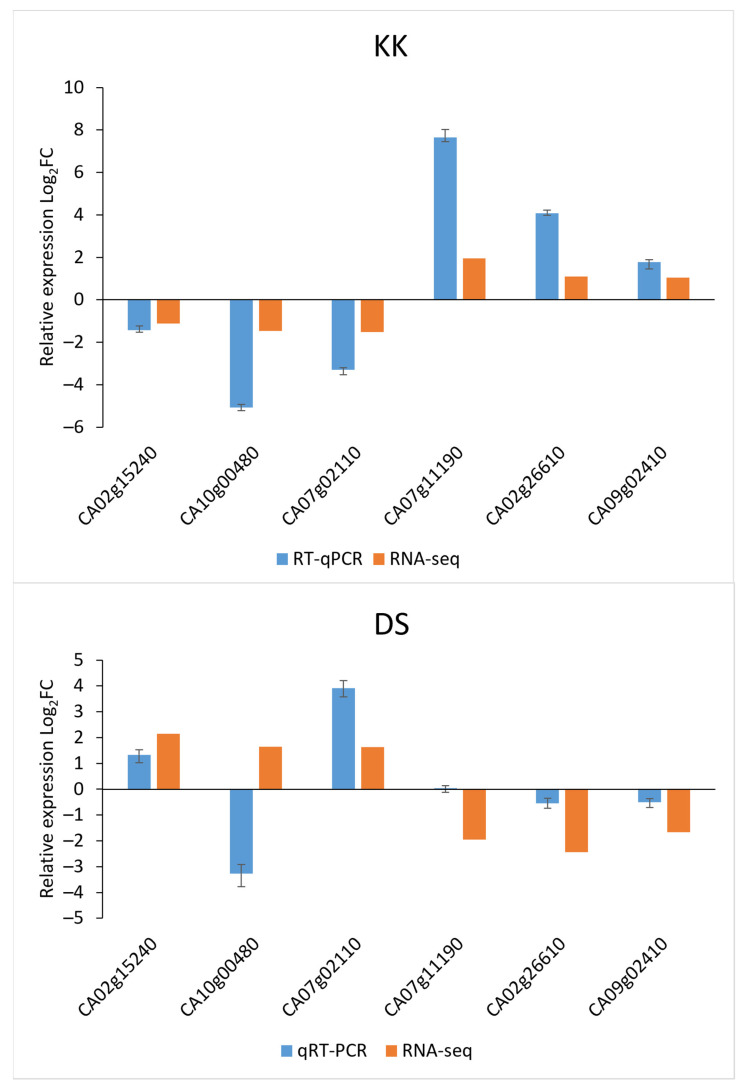
The relative quantification of the expression of selected genes by RT-qPCR and RNA-seq in KK and DS plants infected with PSTVd at 43 dpi.

Among all the overlapping DEGs showing inverse expression patterns in the two cultivars, we selected six genes for validation by quantitative RT-PCR (RT-qPCR). Among these six genes, CA02g15240 (auxin-repressed 12.5 kDa protein-like isoform), CA10g00480 (NADPH: protochlorophyllide oxidoreductase (*POR*)), and CA07g02110 (polygalacturonase-inhibiting protein (*PGIP*)) were downregulated in infected KK plants and upregulated in infected DS plants, whereas CA07g11190 (1-aminocyclopropane-1-carboxylic acid oxidase (*ACO*)), CA02g26610 (S-adenosylmethionine decarboxylase proenzyme (*SAMDC*)), and CA09g02410 (phenylalanine ammonia-lyase (*PAL*) were upregulated in KK plants and downregulated in DS plants upon infection, according to the RNA-seq data. The results of the RT-qPCR analysis confirmed the gene expression profiles in infected KK plants, as it was in RNA-seq, and showed that the CA10g00480, CA02g26610, and CA07g11190 genes exhibited the greatest change in expression. In infected DS plants, two genes (CA10g00480 and CA07g11190) showed different patterns according to the RNA-seq data (Figure 5), and CA10g00480 and CA07g02110 showed the greatest change in expression according to RT-qPCR.

## 3. Discussion

Global gene expression analyses following PSTVd infection have been conducted in various *Solanaceae* plant species (tomato, potato, and potato vine) [27,28,33]. The obtained data have revealed different sets of down- and upregulated genes, depending on the host genotype, viroid strain pathogenicity, and infection stage [22,28,29,34]. The response of pepper to PSTVd infection is still poorly understood, which has encouraged our research in this direction [31].

In the current study, we applied RNA-seq to explore the response of two pepper cultivars to PSTVd infection. We selected the Bulgarian pepper cultivars, Capsicum annuun Djulunska shipka and Kurtovska kapia, as they are the most widely grown hot and sweet pepper cultivars in the country and showed PSTVd- specific phenotypes. Our transcriptome study was the first to characterize the pepper–PSTVd interaction and could provide candidate genes for further functional analysis that might highlight the molecular mechanism of the defense response of pepper cultivars.

Previously, the difference in PSTVd responses between susceptible and tolerant tomato cultivars showed changes in the expression levels of genes involved in the hormone metabolism or the signaling pathway [35]. Hormones are major players in systemic signaling and responses during plant growth and development, and changes in their expression levels affect the phenotype of the plant. Symptoms caused by PSTVd infection, such as epinasty and growth retardation, have been associated with changes in GA, auxin, and BR levels [35]. The role of auxin in PSTVd-induced defense signaling in plants has been reported by several research groups [17,27,33,35]. An altered expression of auxin-related genes, with most genes being downregulated, was found in PSTVd-infected plants of the potato cultivar Solanum tuberosum Safari [27] and the tomato cultivar Solanum lycopersicum Rutgers [29]. The altered level of indole-3-acetic acid (IAA) was associated with the loss of apical dominance in the PSTVd-infected tomato cultivar Solanum lycopersicum Rutgers [36]. Organ-specific changes in endogenous IAA levels were correlated with the expression of genes involved in IAA metabolism in PSTVd-infected tubers of the potato cultivar Solanum tuberosum Désirée [37]. In line with these data, our study found the altered expression of hormone-related genes in PSTVd-infected pepper cultivars. CA02g15240 (auxin-repressed 12.5 kDa protein-like isoform) responds to PSTVd in a cultivar-specific manner, suggesting a hormone-mediated disease control against the viroid (Figure 5). Therefore, the increased expression of CA02g15240 in DS plants might be associated with the higher sensitivity of this cultivar to infection, as exemplified by the more severe disease symptoms (Figure 5).

PSTVd infection altered expression levels of three ethylene biosynthesis genes in tomato: *SAMDC*; aminocyclopropane-1-carboxylic acid synthase (*ACS*); and *ACO*, [29,35]. *SAMDC* showed dynamic expression, whereas the *ACO* gene showed an increase in expression with the progression of the PSTVd infection [29]. The expression of the CA07g11190 (*ACO*) gene was upregulated in infected KK plants (Figure 5), suggesting that PSTVd may enhance ethylene production, which could improve plant stress tolerance. Moreover, *SAMDC* is also a key enzyme involved in the biosynthesis of the main types of polyamines (PAs), which exert complex pleiotropic effects [38,39]. Transgenic potato with reduced PAs, due to downregulated *SAMDC*, displayed a stunted phenotype, small leaves, and inhibited root growth [40]. The stronger phenotype in infected DS is possibly linked to the reduction in *SAMDC*. PAs are regulators of ion homeostasis, which may be related to the DS-specific DEGs enriched in the GO BP terms transmembrane transport and anionic transport. Additionally, in DS we detected several downregulated genes involved in phenylpropanoid and cinnamic acid metabolism according to the GO analysis (Table S4). One of them is an ortholog of *PAL*, which is involved in the metabolism of phenylpropanoids and contributes to the virus resistance by influencing the SA accumulation [41,42]. *CaPAL1*-silenced pepper plants exhibited decreased levels of SA and *CaPR1* gene expression and were more susceptible to *Xanthomonas campestris* pv. *vesicatoria* [43]. Infection with a highly virulent PSTVd strain quickly reduced *PAL* expression in tomato plants [29]. In DS, CA09g02410 (*PAL*) was downregulated (Figure 5), which could underlie the symptom severity upon infection.

Photosynthesis disruption in PSTVd-infected plants has been linked to the suppression of genes involved in chloroplast biogenesis and function, such as *POR3*, *Cab4*, *Cab5*, and chloroplast carbonic anhydrase (*CA*) [34]. Numerous genes involved in the light reaction were downregulated, and the number of downregulated tomato genes varied with the PSTVd strain and the time point post-inoculation [34]. Consistent with the previous report, downregulation of the *POR* gene was detected in both pepper cultivars according to RT-qPCR (Figure 5). In addition, the RNA-seq analysis revealed the reduced mRNA levels of other chlorophyll biosynthesis genes (CA04g17210, CA08g13530, CA05g01250, CA10g22340) and Chl a/b binding proteins (CA02g12070, CA07g10990, CA10g02050, CA03g29950, CA06g18800, CA09g14040) in PSTVd-infected KK and DS (Table S1), which collectively could contribute to the PSTVd symptoms by enhancing chlorosis during later stages.

Many DS-specific DEGs were associated with GO BP term response to stimulus (Table S3, Figure S3). *PGIPs* respond to various stimuli by modifying the expression patterns of genes in several metabolic pathways, which strengthen the cell wall and confer increased resistance against certain fungal pathogens [44,45]. CA07g02110 (*PGIP*) showed an inverse expression pattern in the two analyzed pepper cultivars (Figure 5); therefore, its role in pepper cultivar-specific response should be analyzed further to outline its spatial and temporal induction.

Various TF-encoding genes exhibiting differential expression in PSTVd-infected tomato and potato cultivars have been reported previously [17,27,46]. Our data showed a large number of DE TFs in both infected pepper cultivars (Figure 2b). The most abundant and upregulated DS-specific DE TFs included CA10g14960 (MYB TF), CA04g17920 (C2H2-type zinc finger protein), CA02g04730 (nuclear transcription factor Y subunit A-7-like (NF-YA7-like)), CA02g01120 (Dof3 protein), and CA09g07450 (ethylene-responsive element binding factor (ERF)) (Table S2). In contrast, in the KK cultivar the most altered DE TF genes included CA01g00510 (TT2-like MYB TF), CA09g08120 (WRKY71), CA10g18410 (homeodomain (HD)-related), CA11g12710 (WRKY75-like), CA11g14620 (Myb4-like), CA05g00840 (heat stress transcription factor B-3-like (HsfB3-like)), CA02g05780 (APETALA2 (AP2)/ERF domain-containing TF), and CA03g35110 (DNA-binding protein homolog) (Table S2). Among the overlapping DE TFs, we found an increased expression of the gene CA11g16170 encoding BEL-5 transcript. The upregulation of the potato *BEL-5* homolog was found to affect tuber formation by repressing GA synthesis upon PSTVd infection [27]. Whether there is a role for *BEL-5* in GA metabolism in pepper, and what it is, needs to be further clarified. Twelve overlapping DE TF genes, belonging to eight gene families—i.e., *C2H2* (CA08g16870), *HD-ZIP* (CA08g08650, CA02g07270), *WRKY* (CA09g11950, CA02g01800, CA02g30960), *NAC* (CA05g04410), *NF-YA* (CA10g20490), *bHLH* (CA03g06220, CA04g17180), *HSF* (CA10g20440), and *CO-like* (CA02g26690, CA11g01070)—showed contrasting expression patterns in the two pepper genotypes (Figure S3). Our study reported several DE TFs in the pepper response to PSTVd infection, but the specific role of each of them should be clarified in a further in-depth study.

## 4. Materials and Methods

### 4.1. Plant Material and PSTVd Inoculation

Two Bulgarian pepper cultivars, KK and DS, representing sweet and hot peppers, respectively, were grown in a greenhouse under the standard conditions of 16/8 day/night photoperiod at 23 °C. The plants were mechanically inoculated with PSTVd (accession number X58388) transcript, synthesized in vitro, as previously described, by rubbing onto the second true leaf pre-treated with carborundum. As a mock control, the plants were inoculated with a 1% potassium phosphate buffer pH 7.0. Samples were collected from the upper part of the plants in the late stage of infection (43 dpi). Leaves collected from five separate plants from each experimental group (KKH, KKI, DSH, DSI) were pooled for RNA extraction.

### 4.2. RNA Extraction, cDNA Synthesis, and Illumina Sequencing

The total RNA was extracted using the RNeasy Plant Mini Kit (Qiagen, Valencia, CA, USA). The quality and quantity of RNA were verified using the Epoch™ microplate spectrophotometer (Bad Friedrichshall, Germany) and agarose gel electrophoresis. The RNA samples were shipped on dry ice to Novogene Technology Co., Ltd. (Beijing, China) for cDNA library construction and RNA sequencing using an Illumina HiSeq 2000 platform, where 150 bp paired-end reads were generated. Two libraries (mock (H) and infected (I)) for each cultivar (KK and DS) were prepared and sequenced. Adapter sequences and low-quality reads were filtered from the data by Novogene Technology Co., Ltd. The sequencing datasets for all samples were deposited on the NCBI server under the accession number PRJNA762255 (https://www.ncbi.nlm.nih.gov/bioproject/PRJNA762255 accessed on 6 December 2021).

To synthesize cDNA, 1 µg of total RNA was reverse-transcribed using oligo (dT) primers and the RevertAid First Strand cDNA Synthesis Kit (Thermo Scientific, Waltham, MA, USA).

### 4.3. Identification and Functional Analysis of DEGs

The quality check and adapter trimming of the FASTQ files were preformed using the Galaxy platform (FastQC (Galaxy version 0.73), TrimGalore (Galaxy version 0.6.7)) [47]. The clean reads were mapped to the Zunla 1 v2.0 pepper reference genome in the Galaxy platform using HISAT2 (Galaxy version 2.2.1). The number of counts for gene transcripts was determined based on feature counts, according to the genome annotation. Count matrices were used for the differential expression analysis and were uploaded to the DESeq2 package (Galaxy version 2.11.40) using a negative binomial distribution model [32]. DEGs selected for further analysis were chosen when |log2FC| ≥ 1 and *p* < 0.05. GO enrichment analysis was performed for the selected genes using g: Profiler, a tool available on the g:GOSt server with a g:SCS multiple testing correction method applying a g:SCS significance threshold with a user threshold of 0.05. The enriched GO terms were summarized using REVIGO with default parameters [48,49]. The pepper TFs were downloaded and mapped from PlantRegMap/PlantTFDB v5.0 [50].

### 4.4. Semi-Quantitative RT-PCR and RT-qPCR Analysis

Semi-quantitative RT-PCR was performed in nonsaturating PCR conditions (28 cycles) using cDNA (dilution factor 2, 3, and 4) and primers to amplify the PSTVd (+) strand and the reference gene EF1a. The primers used are listed in Table S5. The PCR protocol was: 94 °C for 2 min, followed by 28 cycles of 94 °C for 30 s, 63 °C for 30 s and 72 °C for 1 min, and final elongation at 72 °C for 5 min.

Three DEGs revealed by high-throughput sequencing were selected for RT-qPCR validation. cDNAs, prepared as described in Section 4.2, were diluted 10 times in water, and 4 μL of the diluted cDNA solution was used as a template in the PCR reaction. Each PCR reaction contained 0.75 μL of each primer (10 μM), 7.5 μL of DNase- and RNase-free water, and 12 μL of 2 × SYBR Low-ROX mix (Genaxxon, Bioscience), for a final volume of 25 μL. The primer pairs used for qPCR are listed in Table S5. All reactions were initiated at 50 °C for 2 min, followed by 95 °C for 10 min with ensuing 40 amplification cycles of 95 °C for 15 s and 60 °C for 1 min. The housekeeping *EF1A* gene was used as a control to normalize the expression of the selected genes. Three biological replicates were tested per each target gene. The relative gene expression was calculated according to the 2^–ΔΔCt^ method.

## 5. Conclusions

Overall, our study showed that the hot and sweet pepper cultivars respond to PSTVd infection with specific changes in gene expression. We found several DEGs associated with the PSTVd-induced response in pepper, which showed contrasting expression patterns in the two cultivars. The cultivar-specific genes involved in photosynthesis, plant hormone signaling, and defense pathways could reshape the pepper cultivar-specific response to infection and disease symptom intensity. Our findings suggest multilayer protection against PSTVd to control systemic infection in pepper cultivars. In the future, strategies for the management of PSTVd infection in pepper plants may be developed based on the molecular characterization of host–viroid interactions, including more cultivars with distinctive PSTVd susceptibility phenotypes.

## Figures and Tables

**Table 1:** Total raw, clean, and mapped reads obtained from each sample.

Sample	Raw Reads	Clean Reads	Mapped Reads
KKH	40,435,192	98.99%	74.64%
KKI	40,171,400	98.22%	73.8%
DSH	49,817,968	98.50%	76.19%
DSI	42,984,488	98.52%	74.08%

KKH, mock KK; KKI, infected KK; DSH, mock DS; and DSI, infected DS.

## Data Availability

The sequencing data are available at NCBI SRA with acc. number: PRJNA762255.

## Supplementary Materials

The following are available online at https://www.mdpi.com/article/10.3390/plants10122687/s1: Figure S1. RT-PCR data for detection of EF1a in KKH, KKI, DSH, DSI (A), and of PSTVd (+) strand in KKH, KKI, DSH, DSI (B); Figure S2. Relative accumulation of PSTVd (+) strand in KKI and DSI, as determined by semi-quantitative RT-PCR; Figure S3. Gene Ontology scattered plot constructed by REVIGO; Table S1. List of DEGs in DS and KK cultivars in response to PSTVd infection; Table S2. List of overlapping DE TFs; Table S3. GO enrichment analysis of overlapping and cultivar-specific DEGs; Table S4. GO enrichment analysis of up- and downregulated DEGs; Table S5. List of primers used in this study.
