# Supplementary material for: Transcriptome Analysis Reveals Dynamic Cultivar-Dependent Patterns of Gene Expression in Potato Spindle Tuber Viroid-Infected Pepper
Source: Plants (Basel). 2021 Dec 7;10(12):2687. doi: 10.3390/plants10122687 (PMC8706270; doi:10.3390/plants10122687)
Supplement: Supplementary file 1 [file plants-10-02687-s001.zip › plants-1428762-supplementary/Figure S3.pptx]

## Slide 1
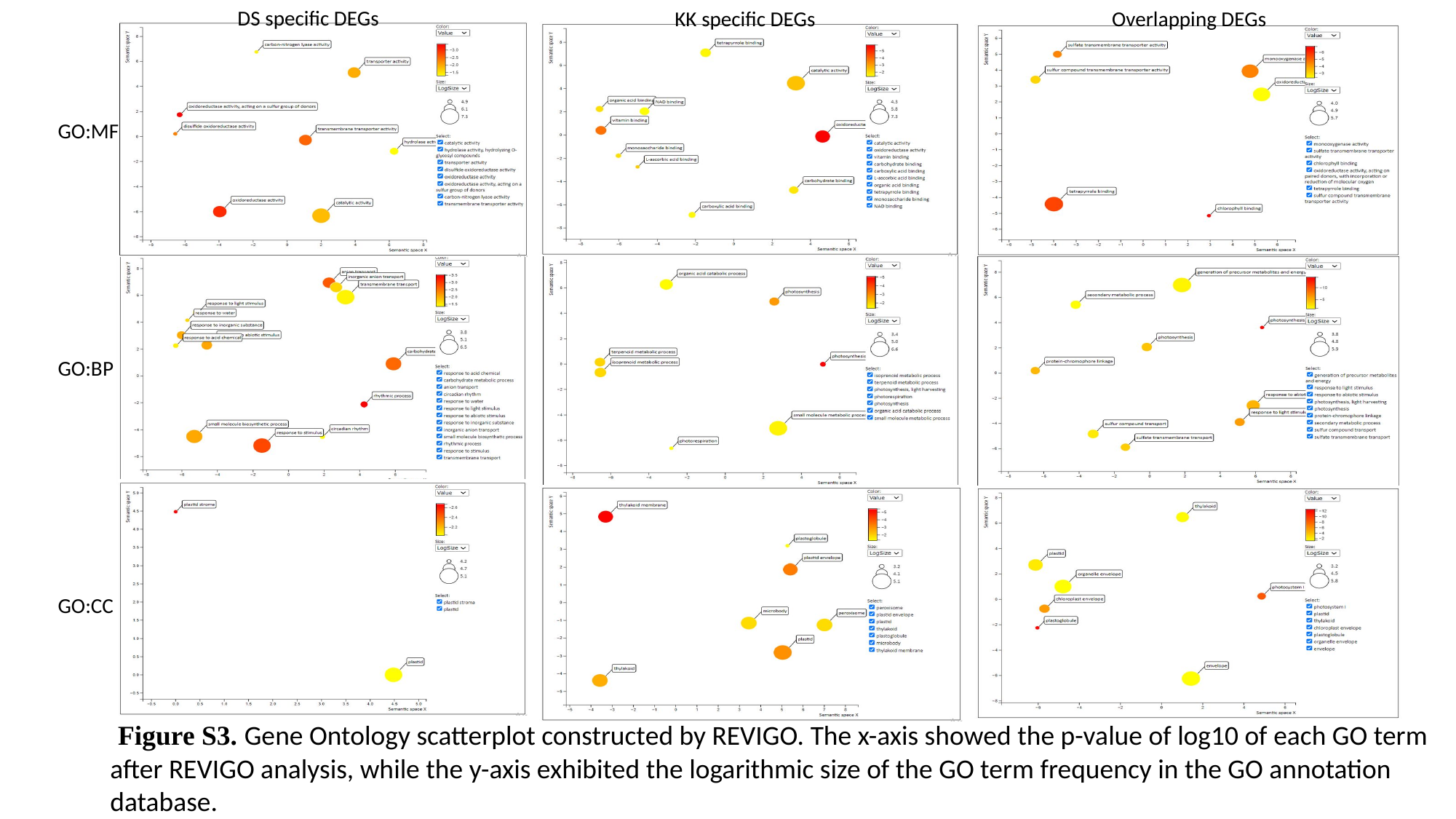

Overlapping DEGs
DS specific DEGs
KK specific DEGs
GO:MF
GO:BP
GO:CC
 Figure S3. Gene Ontology scatterplot constructed by REVIGO. The x-axis showed the p-value of log10 of each GO term after REVIGO analysis, while the y-axis exhibited the logarithmic size of the GO term frequency in the GO annotation database.
