# Supplementary figures and images for: Transcriptome Analysis Reveals Dynamic Cultivar-Dependent Patterns of Gene Expression in Potato Spindle Tuber Viroid-Infected Pepper
Source: Plants (Basel). 2021 Dec 7;10(12):2687. doi: 10.3390/plants10122687 (PMC8706270; doi:10.3390/plants10122687)

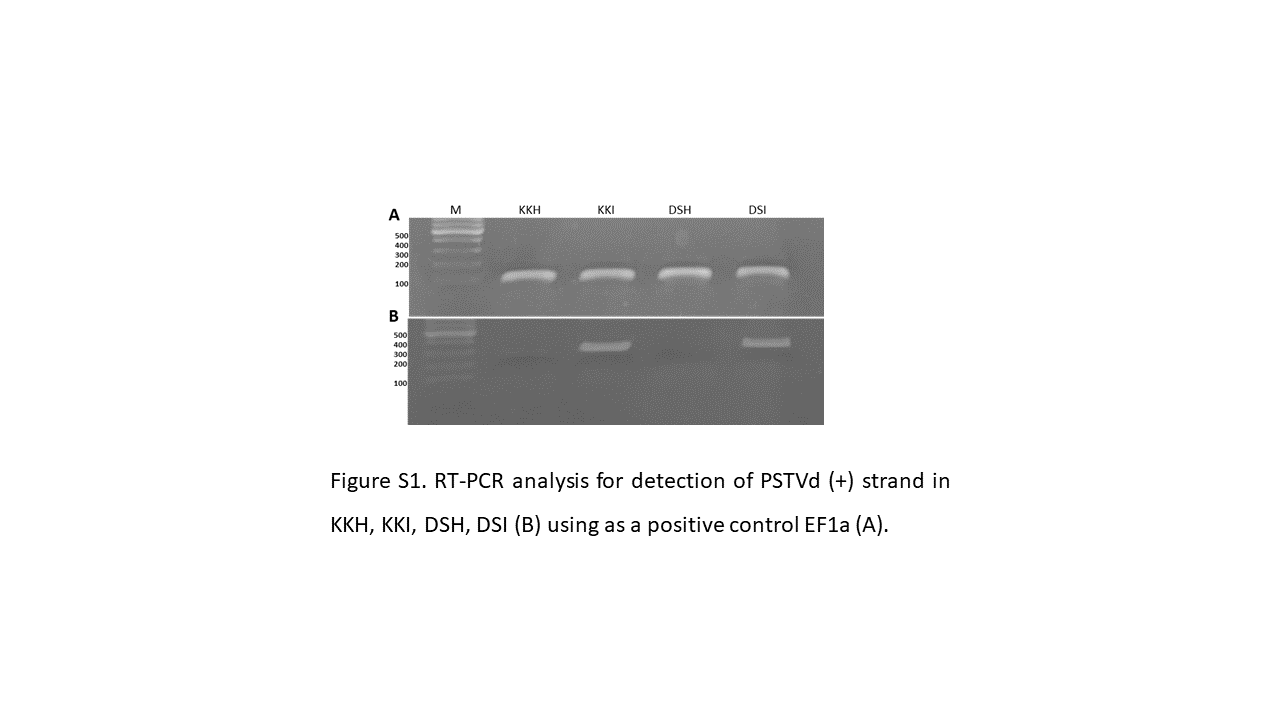

Supplement: Supplementary file 1 [file plants-10-02687-s001.zip › plants-1428762-supplementary/Figure S1.png]

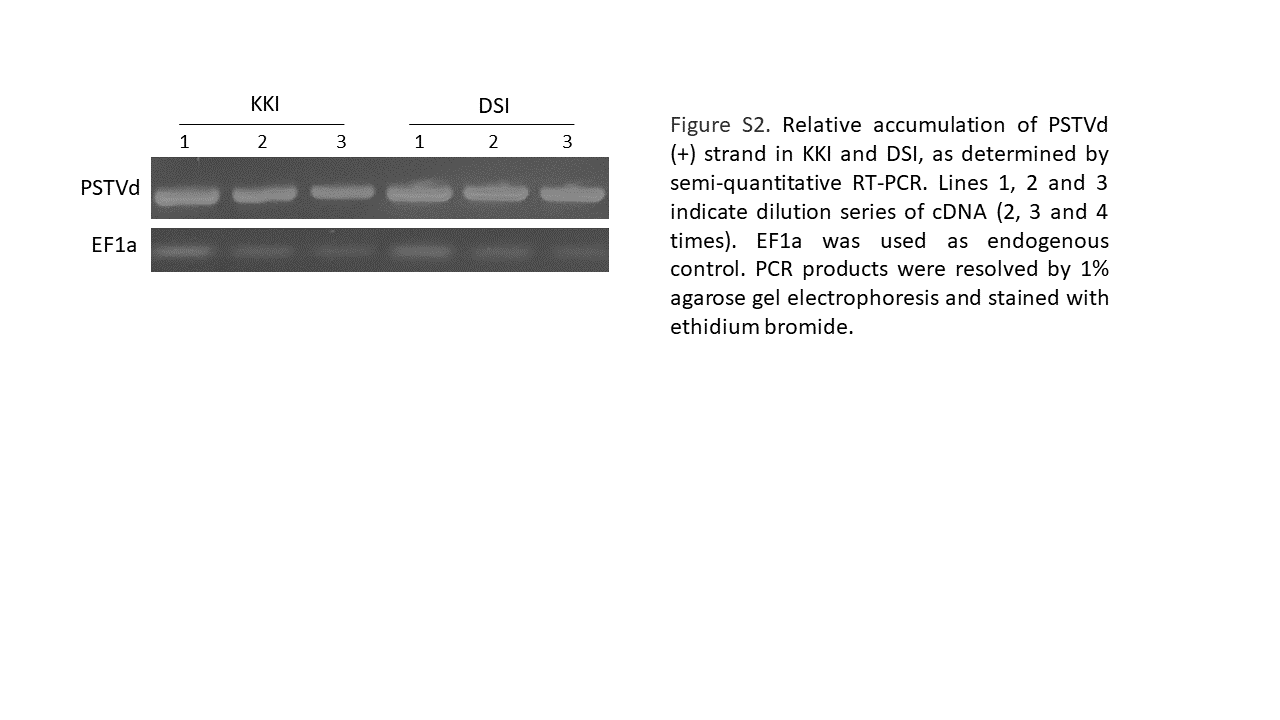

Supplement: Supplementary file 1 [file plants-10-02687-s001.zip › plants-1428762-supplementary/Figure S2.png]
